# Interplay between chronic kidney disease (CKD) and upper tract urothelial carcinomas (UUC): foe or friend?

**DOI:** 10.18632/oncotarget.9753

**Published:** 2016-05-31

**Authors:** Yehong Han, Dawei Shou, Liang Wen, Jianguang Shi, Jian Ding, Ping Gong, Weihua Gong

**Affiliations:** ^1^ Department of Surgery, First People's Hospital of Jiande, Hangzhou City, People's Republic of China; ^2^ Department of Surgery, Second Affiliated Hospital of School of Medicine, Zhejiang University, Hangzhou City, People's Republic of China; ^3^ Department of Thoracic Surgery, Ningbo First Hospital, Ningbo City, People's Republic of China; ^4^ Department of Surgery, Jinan Fourth People's Hospital, Jinan City, People's Republic of China; ^5^ Department of Oncology, First Affiliated Hospital of Shihezi University School of Medicine, Shihezi City, China

**Keywords:** chronic kidney disease, upper tract urothelial carcinomas, association

## Abstract

Although upper tract urothelial carcinomas (UUC) is curable through nephrectomy or nephroureterectomy, progression of chronic kidney disease (CKD) and CKD-related mortality have been highlighted as clinical challenges in recent years owing to the loss of a large number of nephrons. While CKD can promote the development of UUC, other risk factors such as hypertension, diabetes mellitus, advanced age, and anemia can facilitate the progression of CKD. Conversely, CKD is especially prevalent in UUC patients. However, the relationship between CKD and UUC, mechanisms for CKD causing UUC, and gender disparity of UUC of CKD patients have so far not been well-reviewed. As UUC gradually grows, the cancer can be a physical obstacle in the urinary tract. It will cause an increased tract pressure, subsequently resulting in the dysfunction of both nephrons and kidney. At the molecular level, reduced level of oxidative stress was observed in female UUC patients. Furthermore, radical nephrectomy therapy for UUC patients accelerates the progress of chronic kidney dysfunction. Incidentally, the remedies for CKD containing aristolochic acid (AA) are carcinogenic. Our present review offers a comprehensive look at the relationship between CKD and UUC from multiple perspectives. Early and precise identification of progression of CKD and UUC will benefit the patients at high-risk of CKD or UUC, which will also be instructive in directing timely and effective therapeutic interventions whenever necessary. It may also shed light on unveiling the underlying mechanisms of carcinogenesis of UUC, preventing CKD progression, and prolonging the patients' overall survival.

## EPIDEMIOLOGY OF UUC AND CKD

Upper tract urothelial carcinomas (UUC) are sporadic tumors in clinic. However, during chronic renal failure and renal dialysis, the risk of malignancy occurrence particularly urological cancer will be dramatically increased, which was early observed by Matas *et al.* in 1975 [1]. Indeed, UUC including both ureters and kidneys are rare in the Western countries (only about 10% of all renal tumors and 5% of all UUC), whereas in Taiwan a high incidence of UUC was found among urothelial carcinomas cases (up to 20% to 31% of all UUC) [2, 3]. UUC is the most frequently observed urological neoplasm among patients on dialysis in Taiwan. In particular, a significantly higher incidence of UTUC was found in the CKD patients (*p* < 0.001). Co-existence of UUC and uremia can result in an increased morbidity and peri-operative mortality [4, 5]. In return, UUC patients could have an increased incidence rate of kidney function deterioration [6].

Chronic kidney disease (CKD) is common and its prevalence in adults was 10.8% in China [7]. It was especially highlighted that a remarkably high prevalence and incidence of CKD was observed in Taiwan [8]. CKD plays a more influential role than hemodialysis duration in the occurrence of high grade UUC [9]. In patients with CKD or end-stage renal disease (ESRD) an especially higher percentage of high-grade UUC (60%) were observed [4, 9, 10]. Up to 50% UUC bear an aggressive biology developing nodal and distant metastatic disease [11]. High grade UUC is regarded as a more aggressive and invasive cancer [9]. In the Eastern Taiwan, it was found that 58% of pathologically diagnosed patients with UUC had CKD or ESRD [9]. Of note, the clinical pathological features in Taiwan manifested that 60% UUC patients had metastatic or locally advanced diseases upon presentation [2]. Furthermore, multifocal cancers frequently appear in UUC patients [3, 12]. These research findings imply that CKD or ESRD as risk factors might promote the development of urothelial carcinomas especially in the upper urinary tract [9, 13]. Patients with chronic renal insufficiency or kidney transplant recipients were shown to have a high risk of UUC [14]. As the CKD stage of patients advances, the risk of subsequent bladder recurrence will become greater in patients with UUC post-nephroureterectomy [13], and intriguingly vice versa. In 1978, a European study reported that up to 50% of patients with Balkan nephropathy and UUC also suffered from chronic renal insufficiency [15]. The incidence of tumors in the area of Balkan endemic nephropathy was four- to 11-fold increased compared with the neighboring control regions, and up to 60-fold augmented when compared with distant nonendemic areas [16]. In the United States, Lane *et al.* demonstrated that 52% of patients with UUC presented CKD [17]. In Beijing, a 10-year study of 785 UUC patients showed that the proportion of CKD in UUC patients could reach 58.6% and 70.8% in the groups of 70-year age and older [18]. Although only 2% patients with CKD stages 4 and 5 had renal tumor, 15.2% counterparts have UUC [18]. Furthermore, chronic kidney dysfunction could have a higher risk of cancer recurrence. It was previously reported that contralateral upper tract cancer recurrence appeared among 11.3% patients with unilateral UCC after a median 34-month follow-up [2]. In Western countries, acquired renal cell carcinoma has been considered as a complication of long-term dialysis [10].

Therefore, there is great interest in deepening the understanding of the relationship between CKD stages (including ESRD and dialysis) and UUC. Our present article is the first to systematically analyze the progression of CKD-causing UUC, carcinogenic factors, gender disparity of UUC of CKD patients, and chemotherapy for UUC in CKD patients. Furthermore, the prediction of CKD progression or UUC recurrence is also summarized here.

## CURRENT ADVANCES IN EVALUATING CHRONIC RENAL INSUFFICIENCY

Indeed, CKD is a gradual progression of kidney dysfunction. It was suggested that serum creatinine is a standard tool to estimate patients' function [19]. Chronic renal insufficiency was defined as a variation of serum creatinine level between 1.5 mg/dL and 2.0 mg/dL [20]. However, about 5% patients have a normal serum creatinine level but their CKD stage has reached 3 or greater [18]. Therefore, estimated glomerular filtration rate (GFR) is deemed to be a better parameter and is widely employed [9, 18]. Based on estimated GFR, CKD has been divided into five stages. An estimated GFR < 60 mL/min/1.73m^2^ was considered as CKD, while GFR < 15 mL/min/1.73m^2^ or on dialysis is an indication of kidney failure [9].

## MECHANISMS FOR CKD CAUSING UUC

**Figure 1:**
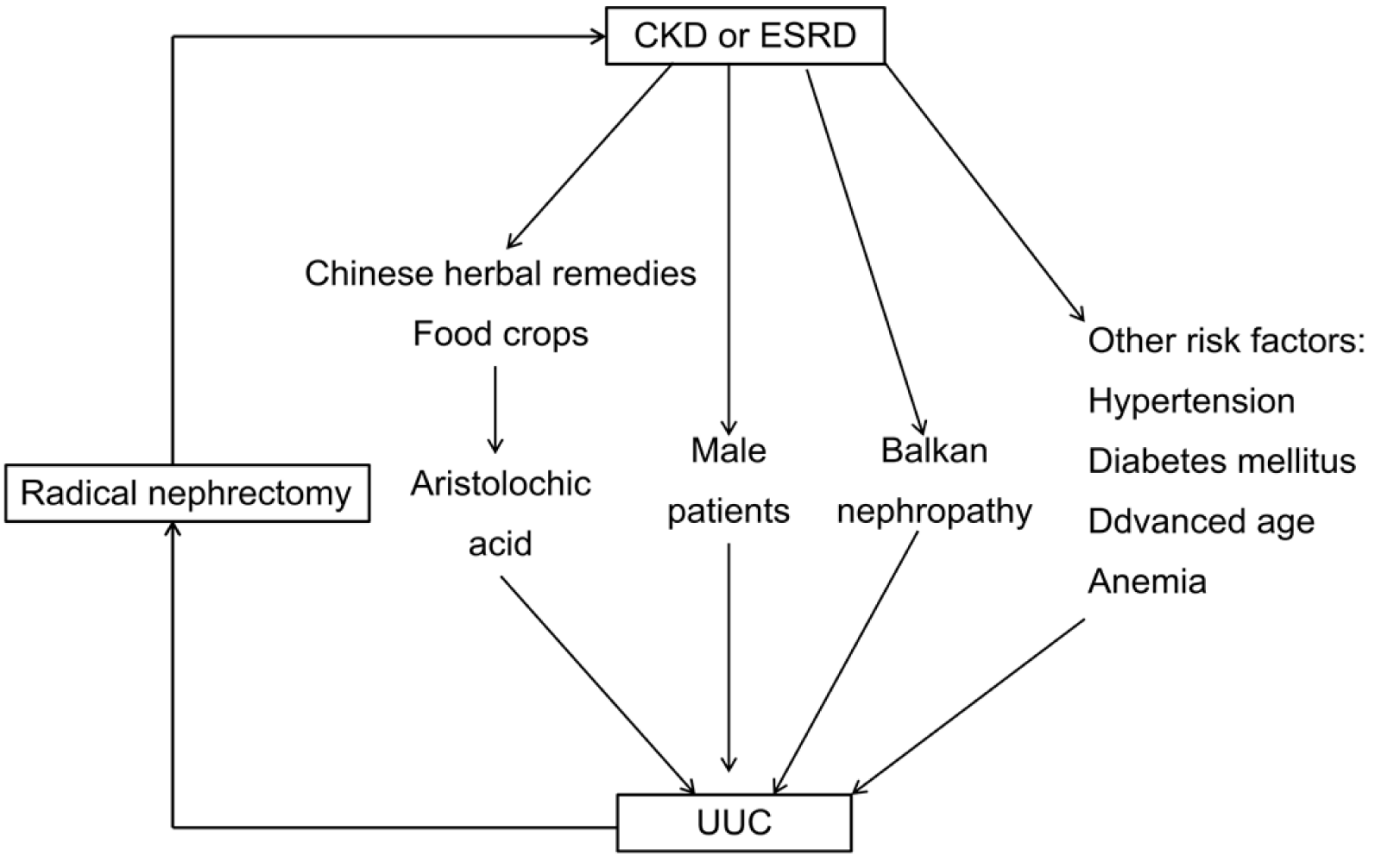
Scheme for relationship between chronic kidney disease (CKD) and upper tract urothelial carcinomas (UUC)

Different pathogenic factors can cause a variety of types of tumors, for instance, mesenchymal tumours in uraemic patients and epithelial and lymphoproliferative cancers in transplant recipients [1]. Indeed, while CKD may play a critical role in the development of urothelial carcinoma particularly the upper urinary tract carcinoma, other risk factors such as hypertension, diabetes mellitus, advanced age, and anemia can facilitate the progression of CKD (Figure 1) [21, 22]. To date, the link between CKD and UUC is insufficiently documented [9]. As UUC gradually grows, in turn, the cancer can be a physical obstacle in the urinary tract. It will cause an increased tract pressure, subsequently resulting in the dysfunction of both nephrons and kidney [18]. On the other hand, most UUC patients received radical nephrectomy therapy which, after surgery, is left with only one functioning kidney. This will, ironically, accelerate the progress of chronic kidney dysfunction [18]. At the molecular level, reduced level of oxidative stress was observed in female UUC patients [21].

Patients with CKD and kidney transplant recipients exhibit a high risk for UUC [14]. A majority of studies on the relationship between ESRD and UUC were from Taiwan and Japan [9]. A high proportion of UUC (47%) was observed among all urinary tract urothelial carcinoma patients with renal failure [3]. It was reported that uremia, which commonly develops with CKD, plays an important role in promoting cancer formation and therefore, renders patients more prone to developing urologic cancer [23]. Uremia has been considered as an independent risk factor for the development of UUC in patients on dialysis [4]. The underlying mechanisms are probably associated with vascular sclerosis, a compromised immune system, and electrolyte imbalance. Narrow therapeutic interval of fluid infusion and poor cardiovascular system (including a heavy blood loss and subsequent blood transfusion) increases the chance for surgical complications to occur, as evidenced by a peri-operative mortality rate of 23.1% among patients undergoing cystectomy [4]. Chronic renal failure could even cause bilateral synchronous and subsequent urothelial tumors of the upper urinary tract [24, 25]. Statistical data from Taiwan showed that 13% of all UUC patients were either metachronous or synchronous cases [2]. A retrospective study on 2,072 patients showed that end-stage analgesic nephropathy (prevalence: 3.1%) could result in a higher risk for urothelial cancers (15.4%, urothelial carcinoma; 9.1%, renal pelvic cancer; and 10.8%, bladder cancer) in comparison to other renal diseases [26]. Further clinical studies exhibited that the incidence of kidney cancer and bladder cancer among chronic hemodialysis patients was 24.1 and 16.4 respectively, significantly higher than that in the general population (*p* < 0.01) [23].

Although the pathogenesis of UUC in ESRD patients remains obscure to date, ‘field cancerization’ theory of carcinogenesis has been proposed based on an observation of a lack of vesicoureteral reflux in dialysis patients. The relatively short duration of haemodialysis procedure, per se, does not lead to malignancy occurrence [27]. Indeed, in Taiwan environmental factors play important roles in the formation of UUC. Nephrotoxic and carcinogenic agents mainly include aristolochic acids (AA) of the Chinese herb owing to such a popularity of Chinese herbs consumption, inducing nephrotubular lesions and malignant neoplastic alteration in the urothelial cells of the entire urinary tract [28]. Nevertheless, further studies on the carcinogens contributing to UUC in patients with CKD are mandatory.

Albeit the underlying mechanisms of UUC formation in the settings of CKD are not well-defined, genetic aberrations play an critical role in tumorigenesis [29]. Genetic investigations indicated pathogenetic influence of chromosomal aberrations among dialysis patients with UUC, as evidenced by losses at 4q, 9p, and 15q and gains at 5p, 7, 19q in UUC of ESRD patients [10]. Gains in these regions were associated with DNA repair genes. It was shown that high-stage and high-grade tumors had more copy number variants [10].

Although the effect of uremia on an increased risk of UUC remains unknown, it is observed that a high prevalence of UUC is found among patients using carcinogenic remedies, such as those containing aristolochic acid (AA) [30]. AA can be responsible for both toxic uremia and UUC [2]. AA, a nitrophenathrene carboxylic acid, originates from plants of the genus Aristolochia, and might inadvertently appear in certain Chinese herbal remedies or food crops (for example, wheat grain contaminated with Aristolochia clematitis seeds) [31-33]. It can rapidly advance the progress of renal interstitial fibrosis [34]. A significant association between AA exposure and an increased risk of UUC was observed in a recent systematic review and meta-analysis [32]. It has been documented that a considerable number of aristolochic acid nephropathy (AAN) cases with concomitant urothelial carcinoma resulted from intake of AA-containing Chinese medicine [34]. As patients are cumulatively exposed to AA, UUC may specifically be induced at a high risk despite its unclear carcinogenetic mechanism [31, 32]. ^32^P-postlabeling assay exhibited that AA-I could bind the active site of cytochromes including P450 1A1 and 1A2, which may partially explain their ability of reductively activating this human carcinogen [35]. AA-DNA adduct was accordingly suggested as a surrogate marker of a risk factor for AAN-associated cancer [36]. Furthermore, AA exposure is capable of inducing mutations in the tumor suppressor gene, TP53, in UUC through A - T transversions on the non-transcribed strand. In other cancers this cluster at hotspots is rarely mutated. The findings clearly depicted a causal association between carcinogen AA and UUC [33]. Cumulative AA exposure was closely associated with end-stage renal disease (ESRD) risk in a dose-dependent manner [32]. In addition, high-arsenic artesian well water is also a contributing risk factor in the development of UUC in Taiwan [9]. Exposure to aforementioned carcinogens can predispose these CKD patients to a life-long risk of UUC including contralateral cancer recurrence (Figure 1) [2].

## GENDER DISPARITY OF UUC OF CKD PATIENTS

Of note, animal studies demonstrated gender disparity in the progression of kidney disease. Male rats demonstrated a more rapid progression of the disease [37]. In line with this, human CKD was also shown to aggravate more rapidly in men than in women (Figure 1) [38]. Furthermore, the incidence of bladder cancer is less common in women (male-to-female ratio: 7:1) in Southern European countries [39]. But female patients have a worse prognosis than males' [40].

However, epidemiological survey in Taiwan showed a higher prevalence of kidney impairment and proteinuria in women than in men [41]. Females had an increased risk of developing CKD [8]. The severity of CKD is closely associated with higher aggressiveness of urothelial cancer [42]. And urothelial carcinogenic mechanisms are probably different between men and women. Female bladder cancer patients are inclined to have more UUC and ESRD compared to males. This close association between UUC and ESRD was merely observed in women but not in men [43]. In Beijing, the incidence of male UUC was significantly lower than that of females' (44.6% *vs*. 55.4%) [18]. Furthermore, UUC in females are characterized by an aggressive histological urothelial carcinoma pattern, as evidenced by the frequency of higher T stage UUC (32.4% and 19.1%), larger UUC (51.4% and 37.8%), and higher grade UUC (89.4% and 75.6%). Paradoxically, they have a more favorable renal outcome [21]. An interesting observation was achieved that in Taiwan male-to-female ratio of primary UUC patients was approximately 2:3, while in other areas the ratio was 3:1 [4]. The Taiwan Cancer Registry reported that the incidence of female UUC was in the range of 3.37 to 4.62 per 100,000 between 1995 and 2003 [43]. In Eastern Taiwan, a study displayed that the incidence of female UUC was a slightly higher than that of males' (male: female = 1:1.16) [9]. This data on the sex ratio of urothelial carcinoma and the ratio of urothelial carcinoma of the renal pelvis to ureter to bladder were apparently different from those of other studies from the Western countries [9], suggesting that a large scale investigation or meta-analysis is required to clarify this issue. Female Taiwanese patients with chronic tubulointerstitial nephritis by Chinese herb nephropathy or analgesic nephropathy are likely to develop UUC [28]. In southern Taiwan, arsenic was found to be an evident carcinogen for the urothelium. UUC is characterized by arsenic-mediated urothelial carcinoma, which is unusually predominant in women [43]. The potential contributing factors of the unusual presentation of female cases need to be further identified and investigated in Taiwan [43]. Of them, some of Chinese herb nephropathy patients received renal transplant therapy, while some were observed to have urothelial carcinoma in the native ureters or kidneys [30].

Balkan endemic nephropathy (BEN) is a chronic tubulointerstitial kidney disease, which is prevalent among the residents along all the tributaries of the Danube river in Bulgaria, Bosnia, Croatia, Herzegovina, Romania, and Serbia [16]. Epidemiological study demonstrated that women with BEN and probable Balkan endemic nephropathy (PBEN) tend more to develop UUC compared to those without Balkan endemic nephropathy (WBEN) (*p* < 0.05). Furthermore, a higher incidence of renal failure was also observed in the BEN (45%) and PBEN (35%) groups (Figure 1) [44]. In addition, it was observed that female UUC of ESRD patients displayed more frequent chromosomal aberrations of chromosome 20q13.2 than their male counterparts [10].

At the molecular level, uremia and dialysis are capable of causing DNA damage. The latter can trigger tumor formation and DNA repair activity is compromised in patients on long term dialysis [10]. Therefore, study on genetic aberration is of great interest. An investigation interestingly revealed that female UUC patients had different genetic profiling, in which considerably more genomic aberrations were found than in males [10]. Several aberrations include 1q22-25.3, 8q, 19p13.2, 20q among amplified chromosomal regions, and 2q21.2-24.3, 9p21.2-24.3, 10q, 11, 13p, 13q14.11, 18p among deleted chromosomal regions in female ESRD patients, while only one aberration 8p12-22 was found in males [10]. This finding may partially explain for the gender disparity in the development of UUC in ESRD patients.

## CHEMOTHERAPY FOR UUC IN CKD PATIENTS

As UUC are urothelial tumors and commonly present as advanced stage and high-grade cancers, cisplatin-based chemotherapy has been routinely proposed [17, 45]. With respect to locally advanced and/or lymph-node-positive UUC, systemic chemotherapy in the neoadjuvant setting was suggested, which can lead to downstaging and complete remission (14-15%) [46, 47]. However, the timing of chemotherapy for UUC is controversial and cisplatin-based chemotherapy is heavily dependent on renal function [48]. The decline in estimated GFR can restrain a substantial number of patients from chemotherapy [17]. The patients' kidney function can be affected by surgical management, as evidenced by a significant decrease (18%) of estimated GFR [48] and a significant increase of the proportion of CKD patients [17] after radical nephroureterectomy (RNU). RNU and bladder cuff excision are per se the gold standard for surgical treatment for UUC [49]. However, Yafi *et al.* reported that 57% patients with good per-operative kidney function were disqualified for cisplatin-based chemotherapy following RNU [11]. The rate of estimated GFR decrease was not dependent of any clinical pathological features [48]. Cisplatin-based chemotherapy requires patients' GFR < 60 mL/min in order to sufficiently excrete the poisonous metabolic waste of chemotherapy [18]. Otherwise, chemotherapy can cause loss of renal units which, in turn, decreases eligibility for systemic chemotherapies [48]. With respect to UUC patients, the deterioration of kidney function post-nephroureterectomy will result in an adverse chemotherapy [50]. Xylinas *et al.* demonstrated that only a small proportion of patients at a high risk of UUC could be subject to adjuvant chemotherapy based on estimated GFR. RNU dramatically decreased the rate of eligibility [48]. Interestingly, 57% of the high-risk patients with normal preoperative kidney function could not become eligible for cisplatin-based chemotherapy after radical nephroureterectomy [11]. Yafi *et al.* demonstrated that adjuvant chemotherapy would not improve patients' survival [11]. Taken together, care should be taken for clinicians to avoid exacerbating the patients' kidney function during the development of comprehensive treatment for UUC [18]. The nephrotoxicity of chemotherapeutics should not be underestimated for the patients older than 70 [17].

## PREDICTION OF CKD PROGRESSION OR UUC RECURRENCE

Although UUC is curable through nephrectomy or nephroureterectomy, post-operation CKD progression and CKD-related mortality have been highlighted as clinical challenges in recent years owing to the large amount of nephrons loss [50]. Deliberate surveillance of remaining functioning renal units is required to early identify preclinical or smaller contralateral recurrent tumors particularly for the progressively increased incidence of asymptomatic UUC [2]. UUC normally have a poor clinical prognosis despite surgical intervention, as evidenced by low 5-year disease-specific survival rate (60%) among high-risk UUC patients [11]. Current guidelines utilize an estimated GFR or albuminuria or abnormal imaging to reflect kidney dysfunction. However, these indicators are not sensitive and specific [19, 50]. To predict CKD progression after nephrectomy or nephroureterectomy, two apoptotic regulatory molecules (Fas and Bcl-2) at mRNA and protein expression levels were examined in surgically resected specimens from 100 patients. These two genes are responsible for regulating apoptosis and involved in tumorigenesis [50]. Research findings revealed that a Fas/beta-actin mRNA ratio > 0.3 and glomerular Fas protein expression were independent prognostic indicators for significantly increased cardiovascular mortality rates and severe renal functional deterioration (SRFD) [50]. At the genetic level, gain of copy number of chromosome 20q13.2 is considered as a causal factor for chromosome instability. Therefore, it was suggested as sole molecular biomarker independently predicting subsequent intravesical recurrence after nephroureterectomy for UUC (*p* = 0.036) [51]. Nevertheless, the aforementioned potential prognostic markers are not ideal. Indeed, the applicable non-invasive biomarkers are required in practice, which provides possibilities of repetitive sampling for patients such as urine or peripheral blood. Furthermore, the identified biomarkers should be independently validated and standardized by mul­tiple international centers [19, 52].

## CONCLUSION REMARKS

With respect to the patients suffering from CKD, cautions should be exercised to minimize intake of Chinese herbs particularly for those with nephrotoxic and carcinogenic agents. Closely monitoring the occurrence of UUC is required irrespective of their dialysis treatment or transplantation therapy. Accurately dynamical prediction and necessary interventions are expected to be explored [19, 52], which will early identify preclinical or asymptomatic UUC. The deliberate surveillance will also offer us more possibilities to unveil the underlying mechanisms of carcinogenesis and prolong the patients' overall survival. Furthermore, gender disparity of UUC of CKD patients should be noted and carefully considered, which will guide our clinical diagnosis and treatment.

Large-scale international scientific studies are needed to determine whether prophylactic nephroureterectomy should be encouraged to perform on one side before transplantation and on the contralateral side after transplantation for the patients with analgesic nephropathy [26]. The role of CKD in tumor recurrence and mortality is required to be further investigated [18]. In addition, apart from radical nephroureterectomy, the role of adjuvant chemotherapy and optimal regimen are to be evaluated without inherent biases of analysis in patients with UUC [11]. To date, care should be taken for clinicians to avoid aggravating the patients' kidney function during the development of comprehensive treatment for UUC. The nephrotoxicity of chemotherapeutics should not be underestimated for the patients older than 70 in clinical practices.
